# Epidemiology of carbapenem-resistant and carbapenemase-producing Enterobacterales in the Netherlands 2017–2019

**DOI:** 10.1186/s13756-022-01097-9

**Published:** 2022-04-09

**Authors:** Cornelia C. H. Wielders, Leo M. Schouls, Sjoukje H. S. Woudt, Daan W. Notermans, Antoni P. A. Hendrickx, Jacinta Bakker, Ed J. Kuijper, Annelot F. Schoffelen, Sabine C. de Greeff, the Infectious Diseases Surveillance Information System-Antimicrobial Resistance (ISIS-AR) Study Group, J. W. T. Cohen Stuart, D. C. Melles, K. van Dijk, A. Alzubaidy, B. F. M. Werdmuller, G. J. Blaauw, B. M. W. Diederen, H. J. Alblas, W. Altorf-van der Kuil, S. M. Bierman, S. C. de Greeff, S. R. Groenendijk, R. Hertroys, E. J. Kuijper, J. C. Monen, D. W. Notermans, W. J. van den Reek, A. F. Schoffelen, A. E. Smilde, C. C. H. Wielders, S. H. S. Woudt, R. E. Zoetigheid, W. van den Bijllaardt, E. M. Kraan, E. E. Mattsson, J. M. da Silva, E. de Jong, B. Maraha, A. J. van Griethuysen, G. J. van Asselt, A. Demeulemeester, B. B. Wintermans, M. van Trijp, A. Ott, J. Sinnige, D. C. Melles, W. Silvis, L. J. Bakker, J. W. Dorigo-Zetsma, K. Waar, A. T. Bernards, M. A. Leversteijn-van Hall, E. Schaftenaar, M. H. Nabuurs-Franssen, H. Wertheim, B. M. W. Diederen, L. Bode, M. van Rijn, S. Dinant, O. Pontesilli, D. S. Y. Ong, M. Wong, A. E. Muller, N. H. Renders, R. G. Bentvelsen, A. G. M. Buiting, A. L. M. Vlek, A. J. Stam, A. Troelstra, I. T. M. A. Overdevest, M. P. A. van Meer, C. Oliveira dos Santos, M. J. H. M. Wolfhagen, the Dutch CPE Surveillance Study Group, A. Maijer-Reuwer, M. A. Leversteijn-van Hall, W. van den Bijllaardt, I. J. B. Spijkerman, K. van Dijk, T. Halaby, B. Zwart, B. M. W. Diederen, A. Voss, J. W. Dorigo-Zetsma, A. Ott, J. H. Oudbier, M. van der Vusse, A. L. M. Vlek, A. G. M. Buiting, L. Bode, S. Paltansing, A. J. van Griethuysen M. den Reijer, M. van Trijp, M. Wong, A. E. Muller, M. P. M. van der Linden, M. van Rijn, M. J. H. M. Wolfhagen, K. Waar, E. Kolwijck, N. al Naiemi, T. Schulin, M. Damen, S. Dinant, S. P. van Mens, D. C. Melles, J. W. T. Cohen Stuart, M. L. van Ogtrop, I. T. M. A. Overdevest, A. P. van Dam, H. Wertheim, B. Maraha, J. C. Sinnige, E. E. Mattsson, R. W. Bosboom, A. Stam, E. de Jong, N. Roescher, E. Heikens, R. Steingrover, A. Troelstra, E. Bathoorn, T. A. M. Trienekens, D. W. van Dam, E. I. G. B. de Brauwer, F. S. Stals

**Affiliations:** 1 Centre for Infectious Disease Control (CIb), National Institute for Public Health and the Environment (RIVM), P.O. Box 1, 3720 BA Bilthoven, The Netherlands; 2 Department of Medical Microbiology, Amsterdam University Medical Centers, Amsterdam, The Netherlands; 3 Department of Medical Microbiology, Leiden University Medical Center, Leiden, The Netherlands

**Keywords:** Enterobacterales, Carbapenem resistance, Carbapenemase production, Surveillance, Risk factors, *E. coli*, *K. pneumoniae*, Travel, Hospitalization

## Abstract

**Background:**

The Netherlands is currently considered a low endemic country for carbapenem-resistant Enterobacterales (CRE) and carbapenemase-producing Enterobacterales (CPE), experiencing only sporadic hospital outbreaks. This study aims to describe susceptibility to carbapenems and the epidemiology of carbapenemase production in Enterobacterales in the Netherlands in 2017–2019.

**Methods:**

Three complementary nationwide surveillance systems are in place to monitor carbapenem susceptibility in the Netherlands. Routine antimicrobial susceptibility test results from medical microbiology laboratories were used to study phenotypic susceptibility of *Escherichia coli* and *Klebsiella pneumoniae*. Pathogen surveillance (of all Enterobacterales species) and mandatory notifications were used to describe the characteristics of CPE positive isolates and affected persons.

**Results:**

The prevalence of isolates with gradient strip test-confirmed elevated meropenem (> 0.25 mg/L) or imipenem (> 1 mg/L) minimum inhibitory concentration (MIC) in the Netherlands was very low in 2017–2019, with percentages of 0.06% in *E. coli* and 0.49% in *K. pneumoniae*, and carbapenem resistances of 0.02% and 0.18%, respectively. A total of 895 unique species/carbapenemase-encoding allele combinations of CPE from 764 persons were submitted between 2017 and 2019, with the annual number of submissions increasing slightly each year. Epidemiological data was available for 660 persons. Screening because of presumed colonisation risk was the reason for sampling in 70.0% (462/660) of persons. Hospitalization abroad was the most common risk factor, being identified in 45.9% of persons.

**Conclusions:**

Carbapenem resistance of *E. coli* and *K. pneumoniae* remains low in the Netherlands. The annual number of CPE isolates slightly increased during the period 2017–2019. Recent hospitalization abroad is the main risk factor for acquisition of CPE.

**Supplementary Information:**

The online version contains supplementary material available at 10.1186/s13756-022-01097-9.

## Background

Carbapenem-resistant Enterobacterales (CRE) and carbapenemase-producing Enterobacterales (CPE) in particular *Klebsiella pneumoniae* and *Escherichia coli*, have been reported all over the world and are the most commonly found microorganisms with resistance to multiple antimicrobials [1, 2]. Carbapenems represent a group of last resort drugs for the treatment of many enterobacterial infections. Therefore, resistance to carbapenems poses significant challenges to clinicians and negatively impacts patient care [1, 2]. CPE can spread easily and are able to colonize and infect patients in healthcare environments and subsequently also in the community. Preventing the transmission of these microorganisms is of major importance and necessitates coordinated international efforts [3–5].

CRE and CPE were first described in Europe in the early 2000’s and their prevalence has since increased [4–6]. The current epidemiology in European countries varies from sporadic imported cases and hospital outbreaks to (inter-)regional spread and CRE and CPE being endemic in healthcare settings [4]. To date, CRE and CPE have mainly posed a problem in hospitals in the Netherlands, though community-associated infections have begun to emerge [2, 5].

In the Netherlands, targeted screening is performed for persons suspected, or at risk of, carrying a highly resistant microorganism (HRMO), such as CPE [7, 8]. Targeted screening is generally performed upon hospital admission following previous hospitalization abroad for > 24 h within the prior two months, or upon transfer from a department in a healthcare institution with an ongoing HRMO outbreak that is not yet under control. In addition, for hospitalized patients previously identified as CPE carriers, follow-up screening is routinely performed.

Three complementary surveillance systems have been implemented in the Netherlands to monitor carbapenem susceptibility and occurrences and outbreaks of CPE. The first system, launched in 2008 and known as the Infectious Diseases Surveillance Information System for Antimicrobial Resistance (ISIS-AR), collects routinely available antimicrobial susceptibility testing (AST) results of all isolates cultured in Dutch medical microbiology laboratories (MMLs) [9]. ISIS-AR is a combined initiative of the Dutch Ministry of Health, Welfare and Sport and the Dutch Society for Medical Microbiology (NVMM). It is coordinated by the RIVM and participation of the MMLs is voluntary [9]. In February 2019, 82% (45/55) of Dutch MMLs were connected to ISIS-AR. In the second system, which was established by the RIVM in 2011, MMLs are requested to submit isolates suspected of producing carbapenemase to the national pathogen surveillance system for molecular typing. A web-based system, Type-Ned CPE, is used to register the isolate and to submit accompanying isolate and patient data. The third system, OSIRIS, is the web-based national notification system. Since 1^st^ July 2019, notification of CPE has become mandatory to control its spread locally, regionally and nationally [7, 10]. The physician requesting the diagnostic test and the MML conducting the diagnosis must both notify the Municipal Health Service (MHS) of persons carrying or infected with CPE. The MHS then notifies the National Institute for Public Health and the Environment (RIVM) via OSIRIS, as defined in the Dutch Public Health Act. This law aims to prevent potential transmission of CPE by enabling the data transfer between the MHS, the MML, the treating physician and involved hospitals and nursing homes.

The current study aims to provide insight into the epidemiology of carbapenem-resistant *E. coli* and *K. pneumoniae* and carbapenemase-producing Enterobacterales in the Netherlands using data from the three different surveillance systems.

## Methods

This study was restricted to the period 2017–2019. Data from 2020 was excluded as it was not representative of the usual epidemiology of carbapenem susceptibility and CPE due to travel restrictions and downscaling of non-urgent healthcare procedures caused by the COVID-19 pandemic.

### Carbapenem susceptibility and prevalence of CRE

The ISIS-AR database was searched for diagnostic isolates (i.e., taken because of a clinical indication) and non-diagnostic isolates (i.e., taken because of increased risk, or surveillance cultures as part of selective digestive tract decontamination (SDD)/selective oropharyngeal decontamination (SOD) at the intensive care unit (ICU), or partial digestive tract decontamination (PDD) in haematology patients) of patients. The search was limited to isolates of the two most prevalent Enterobacterales species, *E. coli* and *K. pneumoniae* isolates, that were sampled in the period 2017–2019 and tested for meropenem and/or imipenem susceptibility by an automated system. Based on the automated minimum inhibitory concentration (MIC), isolates were categorized as having either an:

(i) MIC ≤ the screening breakpoint as defined by the Dutch national guideline (0.25 mg/L for meropenem and 1 mg/L for imipenem) [11];
(ii) MIC > the screening breakpoint and ≤ the EUCAST clinical susceptible (S) breakpoint (2 mg/L for both imipenem and meropenem) (EUCAST version 9.0 [12]);
(iii) MIC > the EUCAST clinical S breakpoint and ≤ the EUCAST clinical resistant (R) breakpoint (8 mg/L for meropenem and 4 mg/L for imipenem) (EUCAST version 9.0 [12]);
(iv) MIC > the EUCAST clinical R breakpoint.

In accordance with the Dutch national guideline recommendations, isolates with automated measured elevated MIC for carbapenems (i.e., MIC > the screening breakpoint) were further investigated using a gradient strip test [11]. Therefore, isolates in ISIS-AR with elevated automated MIC were further investigated for data on gradient strip tests. Only one isolate per patient per species was included across the period 2017–2019. If multiple isolates per patient per species were available, isolates with a gradient strip test were given priority for inclusion over isolates with only an automated test. Furthermore, if more than one of these isolates had a gradient strip test, the most resistant isolate was prioritized for inclusion. The total number of isolates was then calculated per automated MIC categorization as above. Isolates with an elevated automated MIC were further categorized according to the gradient strip test results, using the same categories as above.

### Microbiological characteristics of CPE and genetic clusters

For the national CPE pathogen surveillance, MMLs are requested to submit Enterobacterales isolates with an MIC > 0.25 mg/L for meropenem and/or > 1 mg/L for imipenem [11] and/or producing carbapenemase and/or with a carbapenemase-encoding gene. Since September 2016 on, it has only been possible to submit accompanying isolate and patient data via Type-Ned CPE. This system allows only one isolate per person per Enterobacterales species/carbapenemase-encoding allele (carba-allele) combination within a twelve-month period. As part of the pathogen surveillance, the species is confirmed by MALDI-ToF (Bruker Daltonics GmbH, Bremen, Germany), the MIC for meropenem by gradient strip test, carbapenemase production by carbapenemase inactivation method (CIM) [13], the presence of the predominant carbapenemase-encoding genes by polymerase chain reaction (carba-PCR), and whole genome sequencing (WGS) [14] is performed for all CIM-positive isolates.

Microbiological characteristics were described based on unique CIM-positive species/carba-allele combinations per person for the period 2017–2019. Only the first species/carba-allele combination per person detected during the 3-year period was included. Samples were excluded if they were without a personal identifier or from Dutch Caribbean MMLs.

Genetic clusters were identified for *E. coli*, *K. pneumoniae* complex and *Citrobacter freundii* using whole genome multi-locus sequence typing (wgMLST), and for *Enterobacter cloacae* complex using pan-genome multi-locus sequence typing (pgMLST) [15]. Isolates were considered part of a genetic cluster if their allelic distance was ≤ 25 alleles for *E. coli* or ≤ 20 alleles for *K. pneumoniae* complex, *E. cloacae* complex and *C. freundii* [15, 16]. Clusters were included only if they contained ≥ 2 isolates originating from ≥ 2 persons.

### CPE epidemiological data

Epidemiological data for samples taken between January 2017 and June 2019 were retrieved from the Type-Ned CPE database. From 1^st^ July 2019 onwards, notification of CPE became mandatory, with epidemiological data being collected via OSIRIS. If information overlapped between Type-Ned CPE and OSIRIS, the information from Type-Ned CPE was used. For persons who within the last 2 months before the CPE positive culture had been hospitalized abroad for > 24 h, their reported geographic regions of the world and countries were analysed for the most frequently reported carba-alleles using solely the available Type-Ned CPE data.

### Statistical analysis

Numbers and percentages were calculated for characteristics of CPE isolates/CPE positive persons where applicable. The median and interquartile range (IQR) were calculated for age and the size of the genetic clusters. Microbiological/WGS data on isolates, carba-alleles, and person levels are presented. The data is presented separately for persons with one versus multiple unique CIM-positive species/carba-allele combination(s). The Cochran-Armitage test for trends was used to assess the trends over time, with a *p*-value < 0.05 being considered statistically significant. Descriptive analyses of the epidemiological information were performed per reason for sampling (presumed risk of carriage versus clinical indication). All statistical analyses were performed using SAS version 9.4.

## Results

### Carbapenem susceptibility and prevalence of CRE

**Fig. 1:**
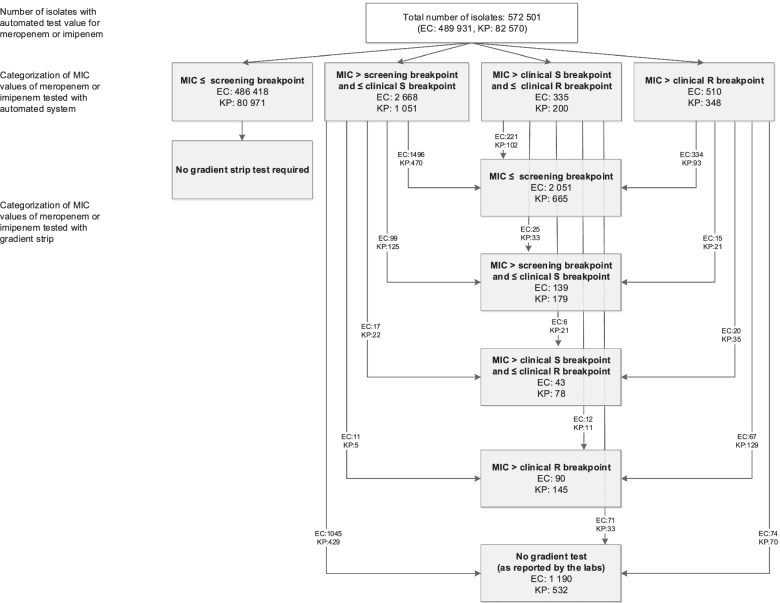
Categorization of automated and gradient strip test results for carbapenem susceptibility in *E. coli* and *K. pneumoniae* between 2017 and 2019 in 43 laboratories participating in the Infectious Diseases Surveillance Information System for Antimicrobial Resistance (ISIS-AR) in the Netherlands. EC: *Escherichia coli*, KP: *Klebsiella pneumoniae*. Screening breakpoint: meropenem 0.25 mg/L, imipenem 1 mg/L (according to the Dutch Society for Medical Microbiology (NVMM) Guideline Laboratory detection of highly resistant microorganisms (HRMO) (version 2.0, 2012) [11]). Clinical S breakpoint: meropenem 2 mg/L, imipenem 2 mg/L (according to EUCAST clinical breakpoint table, version 9.0 [12]). Clinical R breakpoint: meropenem 8 mg/L, imipenem 4 mg/L (according to EUCAST clinical breakpoint table, version 9.0 [12])

From ISIS-AR, routine AST data for 572,501 *E. coli* and *K. pneumoniae* isolates with automated MIC for meropenem and/or imipenem were available from 43 laboratories (covering around 80% of all hospitals/MMLs in the Netherlands) for the period 2017–2019. For automated testing, an elevated MIC was found in 0.9% (5112/572,501) of isolates (Fig. 1). Confirmatory gradient strip testing was performed in 66.3% (3390/5112) of isolates with an elevated MIC (> the screening breakpoint). Gradient strip test-confirmed elevated MIC was found in 12% (272/2323) of the *E. coli* isolates that underwent gradient strip testing and 0.06% of all *E. coli* isolates (n = 489,931). For *K. pneumoniae*, 38% (402/1067) of the isolates that underwent gradient strip testing and 0.49% of all *K. pneumoniae* isolates (n = 82,570) were found to have an elevated MIC. Among the 3513 *E. coli* isolates with an elevated MIC based on automated testing, 133 (3.8%) had an MIC above the clinical S breakpoint for the gradient strip test, of which 90 (2.6%) had an MIC above the clinical R breakpoint. Among the 1599 K*. pneumoniae* isolates with an elevated MIC based on automated testing, these values were 223 (13.9%) and 145 (9.1%), respectively. Thus, gradient strip test-confirmed carbapenem resistance was calculated to be 0.02% in *E. coli* (90/489,931) and 0.18% (145/82,570) in *K. pneumoniae*.

### Microbiological characteristics of CPE and genetic clusters

A total of 895 unique species/carba-allele combinations of CPE (and thus CIM-positive) were detected in isolates submitted to the pathogen surveillance by 50 laboratories for a total of 764 persons (median age = 66 years, IQR = 50–76 years; 53.4% male; Table 1). One unique species/carba-allele combination was detected in 668 persons, whilst 96 persons had multiple isolates (median number of submitted isolates = 2, IQR = 2–2 submitted isolates, range = 2–7 submitted isolates). The median age and sex distribution were similar across the three years. Although not statistically significant, the number of CPE isolates increased from 234 in 2017 to 354 in 2019.

**Table 1:** Number of unique species/carbapenemase-encoding allele combinations^a^ and number of persons^b^ with a CPE isolate cultured in the Netherlands and submitted to the pathogen surveillance system (Type-Ned CPE), 2017–2019

	Number of CPE isolates^a^	Number of persons with CPE^b^	Number of persons with one unique CPE isolate submitted	Number of persons with multiple unique CPE isolates submitted^b^
2017	234	201	172	29
2018	307	264	227	37
2019	354	299	269	30
Total	895	764	668	96

^a^Only one unique species/carba-allele combination was included for the period 2017–2019. When similar unique species/carba-allele combinations were submitted in multiple years, they were only included in the first year in which they were recorded

^b^Persons were only included once for the period 2017–2019. When isolates were submitted for the same person in multiple years, they were only included in the first year in which they were recorded

Of all 895 unique species/carba-allele combinations, 48.2% (431/895) had an MIC for meropenem above the clinical S breakpoint and 30.7% (275/895) had an MIC above the clinical R breakpoint. Subsequently, 51.8% (464/895) unique species/carba-allele combinations had an MIC below or equal to the clinical S breakpoint. An MIC below or equal to the screening breakpoint of 0.25 mg/L for meropenem was observed in 19.2% (172/895). Nevertheless, a carbapenemase-encoding allele was found in 91.8% (158/172), whilst no carbapenemase-encoding allele, despite the positive CIM-test result, was detected in the remaining 14 (8.1%) isolates with an MIC for meropenem below or equal to the screening breakpoint. Nine of those fourteen (64.2%) isolates belonged to the *Enterobacter cloacae* complex. Of the 292 isolates with an MIC for meropenem above the screening breakpoint and below or equal to the clinical S breakpoint (MIC for meropenem > 0.25 and ≤ 2 mg/L), 93.5% (n = 273) had a detectable carbapenemase-encoding allele whilst 6.5% (n = 19) did not. Interestingly, thirteen of those nineteen (68.4%) CIM-positive isolates without a detectable carbapenemase-encoding allele and an MIC for meropenem > 0.25 and ≤ 2 mg/L, belonged to the *Enterobacter cloacae* complex.

Of the 895 total unique species/carba-allele combinations, the most frequently identified species was *K. pneumoniae* complex (39.3%), followed by *E. coli* (34.4%), *E. cloacae* complex (11.3%), and *C. freundii* complex (7.8%; Table 2). For the 227 persons with multiple unique isolates solely, the relative contributions of these species were 35.7%, 33.5%, 10.6%, and 8.8%, respectively (Additional file 1: Table S1). A statistically significant increasing trend was seen for the percentage of urine samples over time (12.4% in 2017 to 19.2% in 2019; *p* = 0.021), and statistically significant decreasing trends were observed for sputum/bronchoalveolar lavage (4.7% in 2017 to 1.1% in 2019; *p* = 0.007) and blood (4.3% in 2017 to 1.4% in 2019; *p* = 0.029; Table 2) over time.

**Table 2:** Species, carbapenemase-encoding allele and material from CPE isolates cultured in the Netherlands and submitted to the pathogen surveillance system (Type-Ned CPE) per year and the trend over time, 2017–2019, the Netherlands

Characteristic	2017	2018	2019	Total	Time trend
	*n* (%)	*n* (%)	*n* (%)	*n*	
Total number of isolates	234	307	354	895	
Total number of carbapenemase-encoding alleles	244	328	379	951	
Species					
* Klebsiella pneumoniae* complex	99 (42.3%)	122 (39.7%)	131 (37.2%)	352	
* Escherichia coli*	82 (35.0%)	90 (29.3%)	136 (38.4%)	308	
* Enterobacter cloacae* complex	31 (13.2%)	38 (12.4%)	32 (9.0%)	101	
* Citrobacter freundii* complex	10 (4.3%)	33 (10.7%)	27 (7.6%)	70	
Other species	12 (5.1%)	24 (7.8%)	28 (7.9%)	64	
Most frequently identified carbapenemase-encoding allele (WGS)					
* bla*_KPC-2_	14 (5.7%)	11 (3.4%)	11 (2.9%)	36	
* bla*_KPC-3_	13 (5.3%)	6 (1.8%)	1 (0.3%)	20	↓
* bla*_NDM-1_	37 (15.2%)	37 (11.3%)	46 (12.1%)	120	
* bla*_NDM-5_	18 (7.4%)	61 (18.6%)	77 (20.3%)	156	↑
* bla*_NDM-7_	5 (2.0%)	6 (1.8%)	10 (2.6%)	21	
* bla*_OXA-48_	89 (36.5%)	125 (38.1%)	145 (38.3%)	359	
* bla*_OXA-181_	13 (5.3%)	22 (6.7%)	26 (6.9%)	61	
* bla*_OXA-244_	6 (2.5%)	10 (3.0%)	11(2.9%)	27	
* bla*_VIM-1_	24 (9.8%)	14 (4.3%)	8 (2.1%)	46	↓
Other carbapenemase-encoding gene	14 (5.7%)	22 (6.7%)	23 (6.1%)	59	
No carbapenemase-encoding gene found	8 (3.3%)	14 (4.3%)	21 (5.5%)	43	
No WGS results	3 (1.2%)	0 (0.0%)	0 (0.0%)	3	
Sample material					
Swab of throat/nose/perineum/rectum	161 (68.8%)	213 (69.4%)	243 (68.6%)	617	
Urine	29 (12.4%)	44 (14.3%)	68 (19.2%)	141	↑
Wound/ulcer/superficial infection	7 (3.0%)	13 (4.2%)	18 (5.1%)	38	
Pus/aspirate/biopsy	10 (4.3%)	4 (1.3%)	8 (2.3%)	22	
Sputum/bronchoalveolar lavage	11 (4.7%)	7 (2.3%)	4 (1.1%)	22	↓
Blood	10 (4.3%)	4 (1.3%)	5 (1.4%)	19	↓
Urine (catheter-related)	3 (1.3%)	9 (2.9%)	3 (0.8%)	15	
Other/unknown	3 (1.3%)	13 (4.2%)	5 (1.4%)	21	

*CPE* carbapenemase-producing *Enterobacterales*, *WGS* whole genome sequencing; ↑ statistically significant increasing trend for the period 2017–2019 (*p* < 0.05); ↓ statistically significant decreasing trend for the period 2017–2019 (*p* < 0.05)

*bla*_OXA-48_ was the most frequently identified carba-allele in CPE isolates overall, as well as in persons with one and with multiple isolates, followed by *bla*_NDM-5_ and *bla*_NDM-1_ (Table 2; Additional file 1: Table S1). A statistically significant increasing trend was observed for *bla*_NDM-5_ over time (7.4% in 2017 to 20.3% in 2019; *p* < 0.001) and statistically significant decreasing trends were observed for *bla*_VIM-1_ (9.8% in 2017 to 2.1% in 2019; *p* < 0.001) and *bla*_KPC-3_ (5.3% in 2017 to 0.3% in 2019; *p* < 0.001; Table 2). A total of 77 genetic clusters with ≥ 2 isolates from ≥ 2 different persons were identified: 36 K*. pneumoniae* complex clusters, 31 *E. coli* clusters, 7 *E. cloacae* complex clusters, and 3 *C. freundii* clusters (Additional files 2 and 3: Tables S2 and S3), with a median size of 2 isolates (IQR = 2–3 isolates, range = 2–38 isolates). Most genetic clusters were caused by *bla*_OXA-48_ (Additional file 2: Table S2). The biggest cluster was caused by a *C. freundii bla*_NDM-5_ outbreak in a hospital (Additional file 3: Table S3). Two or more healthcare facilities were involved in 62.3% (n = 48) of the genetic clusters, 22.1% (n = 17) of the clusters comprised 1 isolate from a hospital and ≥ 1 sample taken by a general practitioner/caregiver at home, 13.0% (n = 10) of the clusters were restricted to 1 healthcare facility, and 2.6% (n = 2) consisted of 2 samples taken by a general practitioner.

### CPE epidemiological data

Accompanying epidemiological data was available for 660 persons (Type-Ned CPE, n = 487; OSIRIS, n = 173). Screening (usually upon admission) because of increased risk for colonisation was the reason for sampling in most persons (70.0%, 462/660; Table 3). Among persons with a positive screening sample, clinical infection due to CPE was reported in 9.1% (42/462), colonisation was reported in 87.0% (402/462), and for 3.9% (18/462) it was unknown whether the patient was colonised or infected. Among persons with a diagnostic isolate, the most common infection was a urinary tract infection (43.8%, 84/192). Half of all CPE positive samples (49.5%) were taken from inpatients, whilst 17.5% of the cases were from outpatients. Of the six predefined risk factors for CPE presence, recent hospitalization abroad was the most frequently reported: 45.9% (303/660) overall, 59.5% (275/462) in persons with a screening sample, and 14.6% (28/192) in those with a diagnostic sample (Table 3). Countries in Western Asia (24.4%, 74/303) and Northern Africa (23.4%, 71/303) were most frequently reported. In 35.2% (232/660) of persons, no risk factor was identified. No major shifts in occurrence of risk factors were observed during the period 2017–2019 (data not shown).

**Table 3:** Epidemiological data of CPE positive persons with an isolate cultured because of a presumed risk for carriage (screening) or a clinical indication (diagnostic) from the pathogen surveillance (Type-Ned CPE; sampling date 1 January 2017–30 June 2019) and of notifications (OSIRIS; sampling date 1 July–31 December 2019), the Netherlands

Characteristic	Reason for culturing		
	Screening (n = 462)^a^	Diagnostic (n = 192)^b^	Total (n = 660)^c^
	*n* (%)	*n* (%)	*n* (%)
Sample taking location^d^			
Inpatient departments (excluding Intensive Care Units)	174/348 (50.0)	67/138 (48.6)	241/487 (49.5)
Outpatient departments	57/348 (16.4)	27/138 (19.6)	85/487 (17.5)
Intensive Care Units	37/348 (10.6)	12/138 (8.7)	49/487 (10.0)
Other/unknown	80/348 (23.0)	32/138 (23.2)	112/487 (23.0)
Residence			
Living independently	378 (81.8)	153 (79.7)	536 (81.2)
Nursing or elderly home/facilities for small-scale housing for elderly	29 (6.3)	21 (10.9)	50 (7.6)
Asylum seekers centre	17 (3.7)	1 (0.5)	18 (2.7)
Rehabilitation centre	5 (1.1)	6 (3.1)	11 (1.7)
Other/unknown	33 (7.1)	11 (5.7)	45 (6.8)
Underlying illness^d^			
No underlying illness	164/348 (47.1)	64/138 (46.4)	228/487 (46.8)
Malignancy/leukaemia or organ/bone marrow transplantation or immunosuppressive therapy (steroids/chemotherapy)	48/348 (13.8)	19/138 (13.8)	68/487 (14.0)
Renal dialysis	13/348 (3.7)	5/138 (3.6)	18/487 (3.7)
Other/unknown	123/348 (35.3)	50/138 (36.2)	173/487 (35.5)
Invasive medical procedure/diagnostics^e^			
No	53/114 (46.5)	28/54 (51.9)	82/173 (47.4)
Surgery	24/114 (21.1)	13/54 (24.1)	39/173 (22.5)
Other (including invasive procedure like endoscopy, cystoscopy, urinary catheter, renal dialysis)	31/114 (27.2)	11/54 (20.4)	43/173 (24.9)
Unknown	6/114 (5.3)	2/54 (3.7)	9/173 (5.2)
Risk factors^f^			
No known risk factor/unknown	94 (20.4)	134 (69.8)	232 (35.2)
Hospitalization abroad for > 24 h during the previous two months	275 (59.5)	28 (14.6)	303 (45.9)
Hospitalized in a country in:			
Western Asia (including Turkey)	64/275 (23.3)	10/28 (35.7)	74/303 (24.4)
Northern Africa	64/275 (23.3)	7/28 (25.0)	71/303 (23.4)
Southern Europe	53/275 (19.3)	4/28 (14.3)	57/303 (18.8)
South Asia	36/275 (13.1)	2/28 (7.1)	38/303 (12.5)
South-eastern Asia	19/275 (6.9)	1/28 (3.6)	20/303 (6.6)
Western Europe	10/275 (3.6)	2/28 (7.1)	12/303 (4.0)
Another region of the world/unknown	29/275 (10.6)	2/28 (7.1)	31/303 (10.2)
Known CPE outbreak in own healthcare facility	26 (5.6)	4 (2.1)	30 (4.6)
Contact with a hospital abroad in the last year in a different way than > 24 h during the previous two months	31 (6.7)	13 (6.8)	45 (6.8)
Already known carrier of CPE	11 (2.4)	3 (1.6)	16 (2.4)
Received care in a department of another healthcare facility with an ongoing outbreak of CPE^g^	14 (3.0)	2 (1.0)	16 (2.4)
Travelling abroad in the past six/twelve months without hospitalization or visiting a hospital^h^	13 (2.8)	8 (4.2)	22 (3.3)

*CPE* carbapenemase-producing *Enterobacterales*

^a^Numbers and percentages are reported on person level with available questionnaire data for the characteristic (n = 462 as denominator) unless otherwise indicated

^b^Numbers and percentages are reported on person level with available questionnaire data for the characteristic (n = 192 as denominator) unless otherwise indicated

^c^Numbers and percentages are therefore reported on person level with n = 660 as denominator unless otherwise indicated. The total number includes 6 persons with an unknown/other reason for culturing in addition to the 462 screening and 192 diagnostic isolates (1 from Type-Ned CPE and 5 from OSIRIS)

^d^This information was only available for the questionnaires in the pathogen surveillance (Type-Ned CPE)

^e^This information was only available for the notification questionnaires in OSIRIS

^f^The total number for this characteristic is higher than the total number of persons presented in the table and the summed percentage is higher than 100% because for some persons more than one answer was registered

^g^Defined in Type-Ned CPE as received care in a department of another healthcare facility with an ongoing outbreak of CPE in the previous two months; defined similarly in OSIRIS but without the addition of “in the previous two months”

^h^Defined as in the past six months in Type-Ned CPE and in the past twelve months in OSIRIS

No predefined risk factor was reported for 69.8% (134/192) of persons with a diagnostic isolate and 20.4% (94/462) of persons with a screening isolate. However, 48.3% (55/114) of persons with a screening isolate notified in OSIRIS underwent an invasive medical procedure or invasive diagnostics prior to detection of the CPE.

When investigating carba-alleles and the geographic regions and countries where CPE positive persons were recently hospitalized, it was observed that *bla*_OXA-48_ was often reported for hospitalizations in Northern Africa and Western Asia, Morocco and Turkey in particular (Table 4). *bla*_NDM_ was often related to countries in Asia, Southern Europe and Northern Africa, whilst *bla*_KPC_ and *bla*_VIM-1_ were more often limited to countries in Southern Europe.

**Table 4:** Most frequently identified carbapenemase-encoding alleles and the corresponding reported geographic regions of the world and countries for persons who were recently hospitalized abroad, January 2017–June 2019^a^, the Netherlands

Carbapenemase-encoding allele (WGS)	Number of persons being hospitalized abroad for > 24 h during the previous two months^b^ (*n*)	Geographic region(s) most frequently reported (*n*)	Most prevalent countries (*n*)
*bla*_OXA-48_	84	Northern Africa (35)	Morocco (29)
		Western Asia (30)	Turkey (29)
		Southern Europe (6)	Egypt (4)
			Spain (4)
*bla*_NDM-1_	37	Southern Europe (9)	Greece (5)
		South-eastern Asia (6)	Turkey (4)
		Northern Africa (5)	India (3)
		Western Asia (5)	Thailand (3)
		South Asia (5)	
*bla*_NDM-5_	29	Southern Asia (12)	India (10)
		Northern Africa (10)	Egypt (7)
			Morocco (3)
*bla*_OXA-181_	22	South Asia (11)	India (7)
		South-eastern Asia (4)	Turkey (4)
		Western Asia (4)	Thailand (3)
*bla*_KPC-2_	15	Southern Europe (8)	Greece (6)
		Western Asia (4)	Turkey (3)
*bla*_OXA-244_	13	Western Asia (6)	Turkey (6)
		Northern Africa (4)	Egypt (3)
*bla*_KPC-3_	12	Southern Europe (11)	Italy (8)
			Portugal (3)
*bla*_VIM-1_	11	Southern Europe (9)	Spain (5)

*CPE* carbapenemase-producing *Enterobacterales*; *WGS* whole genome sequencing

^a^Only data from the pathogen surveillance (Type-Ned CPE) for persons with epidemiological data available were used in this table (sampling date January 2017–June 2019). Recent hospitalization abroad was defined as hospitalized abroad for more than 24 h during the two months prior to the CPE positive culture

^b^Persons with multiple identified carbapenemase-encoding alleles in a single sample were included in these numbers

## Discussion

The prevalence of isolates with gradient strip test-confirmed elevated MIC in the Netherlands was very low in 2017–2019 with percentages of only 0.06% in *E. coli* and 0.49% in *K. pneumoniae*, and carbapenem resistance of 0.02% and 0.18% respectively. The number of CPE isolates submitted to the pathogen surveillance annually increased slightly over the study period, with sporadic clusters mostly confined to a median of two cases in one or two healthcare centres. Recent hospitalization abroad, particularly to the regions of Western Asia and Northern Africa, was identified as the main risk factor for CPE in the Netherlands.

*K. pneumoniae* was the species most often identified as CRE in ISIS-AR and as CPE in the Dutch pathogen surveillance system, followed by *E. coli*. This finding has also been observed in other European countries [17–21]. In addition, the distribution of screening and diagnostic samples (urine being the most important diagnostic sample), was similar to that found in other countries [18–20, 22]. The observed increasing trend in CPE occurrence is in line with previously reported findings in other countries [18–20, 23]. In line with findings from other countries, the most frequently identified carba-allele in CPE isolates in the Netherlands was *bla*_OXA-48_, followed by *bla*_NDM-5_ and *bla*_NDM-1_ [17, 18, 22, 23]. This distribution was also reflected in the genetic clusters that were identified in our study, with most clusters involving *bla*_OXA-48_. In this study, 62.3% (48/77) of the genetic clusters occurred in 2 or more healthcare facilities. Without the use of the pathogen surveillance, most genetic clusters would not have been identified. When there is a multi-institutional cluster, or when a new person is found with an isolate belonging to an already existing cluster, the MMLs are notified and asked to share the identities with other MMLs involved in the genetic cluster [24], in order to detect potential transmission routes and prevent further spread.

Hospitalization abroad during the preceding two months was the main risk factor for CPE in the Netherlands. This finding was also observed in many (mainly Northern European) countries [19, 20, 23]. Turkey and Morocco were the most frequently reported countries, which is not surprising since the majority of Dutch citizens with a migration background originate from these countries [25] and might still often visit relatives there. OXA-48 and OXA-48-like producers are endemic in Morocco and Turkey [3, 26, 27] and acquisition there was also observed in other European countries [18–20]. NDM is endemic in India and Pakistan [3, 26] and has been the cause of outbreaks in Northern Africa and Southern Europe [3, 26]. This corresponds with results from the current study and other studies [18, 20, 28]. KPC is endemic in Italy and Greece [3, 26] which was also reflected in the current study and other studies [18, 20, 28, 29]. Finally, VIM causes significant outbreaks in Southern Europe [26, 27], which explains our findings as well.

For timely identification of nosocomial outbreaks throughout the country, hospitals and long-term care facilities are requested to notify outbreaks of HRMO to the national Early warning and response meeting of Hospital-acquired Infections and AntiMicrobial Resistance (SO-ZI/AMR) group. This expert group, hosted by the RIVM, aims to monitor the course and management of these outbreaks and to analyse and communicate possible risks to public health. From 2017 to 2019, nine CPE outbreaks were reported to SO-ZI/AMR [30]. An *E. coli bla*_VIM-1_ outbreak in an elderly home in 2017 and a *C. freundii bla*_NDM-5_ outbreak in a hospital in 2018–2019 contributed to the significant decreasing trend in *bla*_VIM-1_ and the increasing trend in *bla*_NDM-5_ over time. From the above, it can be concluded that the comprehensive surveillance system provides a true picture of trends in carbapenem resistance among Enterobacterales in the Netherlands.

Thus, a comprehensive surveillance system is essential to monitor carbapenem susceptibility and CPE. Surveillance based only on phenotypical AST results from routine diagnostics may be affected by changes in sampling practices and test performances over time [9]. In our study, 0.9% of isolates had an elevated carbapenem MIC on automated testing. The actual percentage of gradient strip test-confirmed elevated MIC was much lower (0.1%), and this difference is caused by the specificity of the automated systems and possibly the sensitivity of the gradient strip tests. However, confirmatory gradient strip tests are not performed for all isolates with elevated MIC. Besides, the Dutch national guideline was updated in 2021, and confirmatory gradient strip testing is not recommended anymore [31]. Based on a selection of data from ISIS-AR similar to the current study, the percentage of isolates with elevated automated MIC with a gradient strip test performed has slightly decreased, from 70% in 2016 to 65% 2019 in *E. coli* and from 72% in 2016 to 67% in 2019 in *K. pneumoniae*. This is likely compensated by the observed increase in additional tests for carbapenemase production or carbapenemase genes during the period 2015–2019 [30]. Thus, most of the suspected isolates are tested with one or more confirmatory tests: either phenotypically with an MIC measurement and/or CIM test and/or genotypically by PCR. It should be noted that both diagnostic and non-diagnostic isolates were selected from ISIS-AR, and therefore the percentages for elevated MIC and CRE will be lower among infections only. Also, prioritisation of the most resistant isolate might have led to an increase in the reported percentages.

Although the Netherlands has a comprehensive surveillance system, participation is voluntary, data are not always complete, and reconciliation is hard due to the lack of a corresponding identifier. Moreover, only limited information on patient characteristics is available, which complicates drawing conclusions regarding disease burden and transmission. The introduction of mandatory notification of CPE led to more insight into the completeness of the pathogen surveillance: 94.2% of the notifications in our study had a corresponding isolate in the pathogen surveillance. Interestingly, 42 isolates from at least 32 CPE positive persons (the exact number is unknown as no personal identifier was available for 10 samples) were submitted to the pathogen surveillance system without a corresponding notification, potentially caused by differing criteria for isolate submission and notification, or by non-reporting by the MHS or MML.

## Conclusions

Carbapenem resistance for *E. coli* and *K. pneumoniae* remains low in the Netherlands. The predominant CPE species were *E. coli*, *K. pneumoniae* and species belonging to the *E. cloacae* complex. Recent hospitalization abroad was the main risk factor for CPE, with countries in the geographic regions of Western Asia and Northern Africa most often reported. It therefore remains important to perform targeted screening in the Netherlands for persons who have been (hospitalized) abroad recently.

## Supplementary Information

**Additional file 1. Table S1**: Species, carbapenemase-encoding allele1 and material from CPE isolates cultured in the Netherlands and submitted to the pathogen surveillance system (Type-Ned CPE) for persons with one isolate and multiple unique isolates, 2017–2019, the Netherlands.

**Additional file 2. Table S2**: Characteristics of genetic clusters of CPE isolates cultured in the Netherlands and submitted to the pathogen surveillance system (Type-Ned CPE) in the period 2017–2019, consisting of at least two isolates from at least two persons.

**Additional file 3. Table S3**: Detailed list of genetic clusters of CPE isolates cultured in the Netherlands and submitted to the pathogen surveillance system (Type-Ned CPE) in the period 2017–2019, consisting of at least two isolates from at least two persons.

## Data Availability

Data are generated in the process of routine clinical diagnostics and are part of patient medical records. Dutch legislation prohibits that these data be made publicly available. Even if patient identifiable information were to be excluded from the dataset, the very low prevalence of specific HRMO could lead to identification of patients infected with these HRMO. Only researchers from the RIVM have access to the databases. External researchers can submit a data request to the corresponding author, which will subsequently be considered in accordance with the regulations and partnership agreements of the specific surveillance systems.

## Abbreviations

AST: Antimicrobial susceptibility testing
CIb: Centre for Infectious Disease Control
carba-PCR: Carbapenemase-encoding genes by polymerase chain reaction
CIM: Carbapenemase inactivation method
CPE: Carbapenemase-producing Enterobacterales
CRE: Carbapenem-resistant Enterobacterales
EUCAST: European Committee on Antimicrobial Susceptibility Testing
HRMO: Highly resistant microorganism
ICU: Intensive care unit
ISIS-AR: Infectious Diseases Surveillance Information System-Antimicrobial Resistance
MHS: Municipal Health Service
MIC: Minimum inhibitory concentration
MML: Medical microbiology laboratory
NVMM: Dutch Society for Medical Microbiology
PDD: Partial digestive tract decontamination
pgMLST: Pan-genome multi-locus sequence typing
RIVM: National Institute for Public Health and the Environment
SDD: Selective digestive tract decontamination
SOD: Selective oropharyngeal decontamination
SO-ZI/AMR: National Early warning and response meeting of Hospital-acquired Infections and AntiMicrobial Resistance
ST: Sequence type
wgMLST: Whole genome multi-locus sequence typing
WGS: Whole genome sequencing
