# Supplementary material for: Epidemiology of carbapenem-resistant and carbapenemase-producing Enterobacterales in the Netherlands 2017–2019
Source: Antimicrob Resist Infect Control. 2022 Apr 9;11:57. doi: 10.1186/s13756-022-01097-9 (PMC8994189; doi:10.1186/s13756-022-01097-9)
Supplement: Supplementary file 1 — Additional file 1. Table S1: Species, carbapenemase-encoding allele1 and material from CPE isolates cultured in the Netherlands and submitted to the pathogen surveillance system (Type-Ned CPE) for persons with one isolate and multiple unique isolates, 2017–2019, the Netherlands. [file 13756_2022_1097_MOESM1_ESM.docx]

**Additional file 1**

**Table S1.** Species, carbapenemase-encoding allele^1^ and material from CPE isolates cultured in the Netherlands and submitted to the pathogen surveillance system (Type-Ned CPE) for persons with one isolate and multiple unique isolates, 2017–2019, the Netherlands.

| **Characteristic** | **Number of isolates from persons with one unique CPE isolate submitted** | **Number of isolates from persons with multiple unique CPE isolates submitted**^1^ |
| --- | --- | --- |
|  | ***n* (%)** | ***n* (%)** |
| Total number of isolates | 668 | 227 |
| **Species** |  |  |
| *Klebsiella pneumoniae* complex | 271 (40.6) | 81 (35.7) |
| *Escherichia coli* | 232 (34.7) | 76 (33.5) |
| *Enterobacter cloacae* complex | 77 (11.5) | 24 (10.6) |
| *Citrobacter freundii* complex | 50 (7.5) | 20 (8.8) |
| Other species | 38 (5.7) | 26 (11.5) |
| **Most frequently identified carbapenemase-encoding allele (WGS)**^2^ |  |  |
| *bla*_KPC-2_ | 22 (3.1) | 14 (5.8) |
| *bla*_KPC-3_ | 20 (2.8) | 0 (0.0) |
| *bla*_NDM-1_ | 82 (11.6) | 38 (15.6) |
| *bla*_NDM-5_ | 116 (16.4) | 40 (16.5) |
| *bla*_NDM-7_ | 12 (1.7) | 9 (3.7) |
| *bla*_OXA-48_ | 258 (36.4) | 101 (41.6) |
| *bla*_OXA-181_ | 47 (6.6) | 14 (5.8) |
| *bla*_OXA-244_ | 26 (3.7) | 1 (0.4) |
| *bla*_VIM-1_ | 34 (4.8) | 12 (4.9) |
| Other carbapenemase-encoding gene | 47 (6.6) | 12 (4.9) |
| No carbapenemase-encoding gene found | 41 (5.8) | 2 (0.8) |
| No WGS results | 3 (0.4) | 0 (0.0) |
| **Sample material** |  |  |
| Swab of throat/nose/perineum/rectum | 440 (65.9) | 177 (78.0) |
| Urine | 124 (18.6) | 17 (7.5) |
| Wound/ulcer/superficial infection | 30 (4.5) | 8 (3.5) |
| Pus/aspirate/biopsy | 16 (2.4) | 6 (2.6) |
| Sputum/bronchoalveolar lavage | 16 (2.4) | 6 (2.6) |
| Blood | 14 (2.1) | 5 (2.2) |
| Urine (catheter-related) | 13 (2.0) | 2 (0.9) |
| Other/unknown | 15 (2.3) | 6 (2.6) |

CPE: carbapenemase-producing Enterobacterales; WGS: whole genome sequencing.

^1^ Based on unique species/carbapenemase-encoding allele (carba-allele) combinations. Median number of isolates per person was 2, range 2–7.

^2^ Of the persons with a single CPE isolate, 40 had two different carba-alleles in their isolate and therefore a denominator of 708 was used to calculate percentages. Of the persons with multiple CPE isolates, 16 had two different carba-alleles in their isolate and therefore a denominator of 243 was used to calculate percentages for these persons.
