# Supplementary material for: Epidemiology of carbapenem-resistant and carbapenemase-producing Enterobacterales in the Netherlands 2017–2019
Source: Antimicrob Resist Infect Control. 2022 Apr 9;11:57. doi: 10.1186/s13756-022-01097-9 (PMC8994189; doi:10.1186/s13756-022-01097-9)
Supplement: Supplementary file 2 — Additional file 2. Table S2: Characteristics of genetic clusters of CPE isolates cultured in the Netherlands and submitted to the pathogen surveillance system (Type-Ned CPE) in the period 2017–2019, consisting of at least two isolates from at least two persons. [file 13756_2022_1097_MOESM2_ESM.docx]

**Additional file 2**

**Table S2.** Characteristics of genetic clusters of CPE isolates cultured in the Netherlands and submitted to the pathogen surveillance system (Type-Ned CPE) in the period 2017–2019, consisting of at least two isolates from at least two persons.

| **Characteristic** | **Total** | ***K. pneumoniae* complex** | ***E. coli*** | ***E. cloacae* complex** | ***C. freundii*** |
| --- | --- | --- | --- | --- | --- |
| Total number of clusters | 77 | 36 | 31 | 7 | 3 |
| Total number of isolates | 279 | 103 | 102 | 31 | 43 |
| **Number of isolates in genetic cluster** |  |  |  |  |  |
| Median number | 2 | 2 | 2 | 2 | 3 |
| Interquartile range | 2–3 | 2–3 | 2–3 | 2–8 | 2–38 |
| Range | 2–38 | 2–7 | 2–12 | 2–11 | 2–38 |
| **Number of genetic clusters by predominant carbapenemase-encoding allele (WGS)^1^** |  |  |  |  |  |
| *bla*_OXA-48_ | 34 | 18 | 11 | 4 | 1 |
| *bla*_NDM-5_ | 11 | - | 10 | - | 1 |
| *bla*_NDM-1_ | 5 | 5 | - | - | - |
| *bla*_KPC-3_ | 3 | 3 | - | - | - |
| *bla*_OXA-181_ | 3 | 1 | 2 | - | - |
| *bla*_OXA-244_ | 3 | - | 3 | - | - |
| *bla*_VIM-1_ | 3 | - | 1 | 2 | - |
| *bla*_KPC-2_ | 2 | 2 | - | - | - |
| *bla*_OXA-232_ | 2 | 2 | - | - | - |
| *bla*_NDM-7_ | 2 | - | 2 | - | - |
| *bla*_NDM-5_+*bla*_OXA-48_ | 2 | 2 | - | - | - |
| *bla*_NDM-19_ | 1 | - | 1 | - | - |
| *bla*_OXA-514_ | 1 | - | - | - | 1 |
| *bla*_NDM-1_+*bla*_OXA-48_ | 1 | 1 | - | - | - |
| *bla*_NDM-1_+*bla*_OXA-232_ | 1 | 1 | - | - | - |
| *bla*_NDM-5_+*bla*_OXA-181_ | 1 | 1 | - | - | - |
| *bla*_NDM-5_+*bla*_OXA-244_ | 1 | - | 1 | - | - |
| No carbapenemase-encoding gene found | 1 | - | - | 1 | - |
| **Number of genetic clusters by sequence type (STs)** |  |  |  |  |  |
| 10 | 1 | - | 1 | - | - |
| 11 | 1 | 1 | - | - | - |
| 13 | 1 | 1 | - | - | - |
| 15 | 4 | 4 | - | - | - |
| 16 | 3 | 3 | - | - | - |
| 19 | 1 | - | - | - | 1 |
| 22 | 1 | - | - | - | 1 |
| 38 | 7 | - | 7 | - | - |
| 66 | 1 | - | - | 1 | - |
| 69 | 2 | - | 2 | - | - |
| 78 | 3 | - | - | 3 | - |
| **Number of genetic clusters by sequence type (STs)** |  |  |  |  |  |
| 101 | 2 | 2 | - | - | - |
| 121 | 1 | - | - | 1 | - |
| 127 | 1 | - | 1 | - | - |
| 131 | 1 | - | 1 | - | - |
| 147 | 6 | 6 | - | - | - |
| 152 | 1 | 1 | - | - | - |
| 167 | 6 | - | 6 | - | - |
| 227 | 1 | - | 1 | - | - |
| 258 | 1 | 1 | - | - | - |
| 259 | 1 | - | - | - | 1 |
| 294 | 1 | 1 | - | - | - |
| 307 | 6 | 6 | - | - | - |
| 340 | 1 | 1 | - | - | - |
| 354 | 1 | - | 1 | - | - |
| 361 | 1 | - | 1 | - | - |
| 383 | 1 | 1 | - | - | - |
| 391 | 1 | 1 | - | - | - |
| 392 | 1 | 1 | - | - | - |
| 405 | 3 | - | 3 | - | - |
| 410 | 2 | - | 2 | - | - |
| 512 | 1 | 1 | - | - | - |
| 560 | 1 | 1 | - | - | - |
| 591 | 1 | - | - | 1 | - |
| 648 | 1 | - | 1 | - | - |
| 928 | 1 | - | - | 1 | - |
| 940 | 1 | - | 1 | - | - |
| 985 | 1 | 1 | - | - | - |
| 1193 | 1^2^ | - | - | 1^2^ | - |
| 1284 | 1 | - | 1 | - | - |
| 1598 | 1 | - | 1 | - | - |
| 1824 | 1 | 1 | - | - | - |
| 2096 | 2 | 2 | - | - | - |
| 2851 | 1 | - | 1 | - | - |

CPE: carbapenemase-producing Enterobacterales; ST: sequence type; WGS: Whole genome sequencing.

^1^ Sometimes one or more isolates in a genetic cluster had a combination of two different carbapenemase-encoding alleles (carba-alleles). Numbers of genetic clusters in this table are presented according to the predominant identified carba-allele (>50% of the isolates belonging to a cluster). If 50% of the isolates belonging to a genetic cluster had one carba-allele identified, and the other 50% had two different carba-alleles, the cluster was counted as a cluster consisting of isolates with two different carba-alleles.

^2^ One *E. cloacae* complex cluster consisted of ten ST78 isolates and one ST1193 isolate.
