# Supplementary material for: Epidemiology of carbapenem-resistant and carbapenemase-producing Enterobacterales in the Netherlands 2017–2019
Source: Antimicrob Resist Infect Control. 2022 Apr 9;11:57. doi: 10.1186/s13756-022-01097-9 (PMC8994189; doi:10.1186/s13756-022-01097-9)
Supplement: Supplementary file 3 — Additional file 3. Table S3: Detailed list of genetic clusters of CPE isolates cultured in the Netherlands and submitted to the pathogen surveillance system (Type-Ned CPE) in the period 2017–2019, consisting of at least two isolates from at least two persons. [file 13756_2022_1097_MOESM3_ESM.docx]

**Additional file 3**

**Table S3.** Detailed list of genetic clusters of CPE isolates cultured in the Netherlands and submitted to the pathogen surveillance system (Type-Ned CPE) in the period 2017–2019, consisting of at least two isolates from at least two persons.

| **Cluster** | **Species** | **Sequence type (ST)** | **Carbapenemase-encoding allele (WGS)** | **Number of isolates/persons** | **Healthcare facilities involved** |
| --- | --- | --- | --- | --- | --- |
| 1 | *K. pneumoniae* complex | 11 | *bla*_NDM-1_ | 3 | 1 isolate from a hospital and 2 samples taken by a GP |
| 2 | *K. pneumoniae* complex | 13 | *bla*_KPC-3_ | 2 | 2 healthcare facilities |
| 3 | *K. pneumoniae* complex | 15 | *bla*_OXA-48_ | 2 | 1 healthcare facility |
| 4 | *K. pneumoniae* complex | 15 | *bla*_NDM-1_+*bla*_OXA-232_ / *bla*_NDM-1_ | 3 (2 *bla*_NDM-1_+*bla*_OXA-232_ /  1 *bla*_NDM-1_) | 1 healthcare facility |
| 5 | *K. pneumoniae* complex | 15 | *bla*_OXA-48_ | 2 | 1 healthcare facility |
| 6 | *K. pneumoniae* complex | 15 | *bla*_NDM-1_ | 2 | 2 healthcare facilities |
| 7 | *K. pneumoniae* complex | 16 | *bla*_OXA-48_ | 2 | 2 healthcare facilities |
| 8 | *K. pneumoniae* complex | 16 | *bla*_OXA-48_ | 2 | 2 healthcare facilities |
| 9 | *K. pneumoniae* complex | 16 | *bla*_NDM-5_+*bla*_OXA-181_ | 3 (2 *bla*_NDM-5_+*bla*_OXA-181_ /  1 *bla*_OXA-181_) | ≥2 healthcare facilities |
| 10 | *K. pneumoniae* complex | 101 | *bla*_OXA-48_ | 3 | ≥2 healthcare facilities |
| 11 | *K. pneumoniae* complex | 101 | *bla*_OXA-48_ | 2 | 1 healthcare facility |
| 12 | *K. pneumoniae* complex | 147 | *bla*_NDM-1_ | 2 | 1 isolate from a hospital and 1 sample taken by a GP |
| 13 | *K. pneumoniae* complex | 147 | *bla*_OXA-48_ | 2 | 2 healthcare facilities |
| 14 | *K. pneumoniae* complex | 147 | *bla*_OXA-48_ | 2 | 1 healthcare facility |
| 15 | *K. pneumoniae* complex | 147 | *bla*_NDM-5_+*bla*_OXA-48_ / *bla*_NDM-5_ | 5 (4 *bla*_NDM-5_+*bla*_OXA-48_ /  1 *bla*_NDM-5_) | ≥2 healthcare facilities |
| 16 | *K. pneumoniae* complex | 147 | *bla*_OXA-48_ / *bla*_NDM-1_+*bla*_OXA-48_ | 4 (2 *bla*_OXA-48_ /  2 *bla*_NDM-1_+*bla*_OXA-48_) | ≥2 healthcare facilities |
| 17 | *K. pneumoniae* complex | 147 | *bla*_NDM-1_ | 2 | 2 healthcare facilities |
| 18 | *K. pneumoniae* complex | 152 | *bla*_NDM-1_ | 3 | ≥2 healthcare facilities |
| 19 | *K. pneumoniae* complex | 258 | *bla*_KPC-2_ | 3 | ≥2 healthcare facilities |
| 20 | *K. pneumoniae* complex | 294 | *bla*_OXA-48_ | 2 | 1 isolate from a hospital and 1 sample taken by a GP |
| 21 | *K. pneumoniae* complex | 307 | *bla*_OXA-48_ | 4 | ≥2 healthcare facilities |
| 22 | *K. pneumoniae* complex | 307 | *bla*_OXA-48_ | 2 | 2 healthcare facilities |
| 23 | *K. pneumoniae* complex | 307 | *bla*_OXA-48_ | 2 | 2 healthcare facilities |
| 24 | *K. pneumoniae* complex | 307 | *bla*_KPC-3_ | 7 | 1 healthcare facility |
| 25 | *K. pneumoniae* complex | 307 | *bla*_OXA-48_ | 5 | ≥2 healthcare facilities |
| 26 | *K. pneumoniae* complex | 307 | *bla*_OXA-48_ | 4 | ≥2 healthcare facilities |
| 27 | *K. pneumoniae* complex | 340 | *bla*_OXA-181_ | 2 | 1 isolate from a hospital and 1 sample taken by a caregiver at home |
| 28 | *K. pneumoniae* complex | 383 | *bla*_NDM-5_+*bla*_OXA-48_ | 5 | 1 healthcare facility |
| 29 | *K. pneumoniae* complex | 391 | *bla*_OXA-48_ | 2 | 2 healthcare facilities |
| 30 | *K. pneumoniae* complex | 392 | *bla*_OXA-48_ | 3 | ≥2 healthcare facilities |
| 31 | *K. pneumoniae* complex | 512 | *bla*_KPC-3_ | 2 | 1 isolate from a hospital and 1 sample taken by a GP |
| 32 | *K. pneumoniae* complex | 560 | *bla*_KPC-2_ | 2 | 2 healthcare facilities |
| 33 | *K. pneumoniae* complex | 985 | *bla*_OXA-48_ | 5 | ≥2 healthcare facilities |
| 34 | *K. pneumoniae* complex | 1824 | *bla*_OXA-48_ | 2 | 2 healthcare facilities |
| 35 | *K. pneumoniae* complex | 2096 | *bla*_OXA-232_ | 3 | ≥2 healthcare facilities |
| 36 | *K. pneumoniae* complex | 2096 | *bla*_OXA-232_ | 2 | 2 healthcare facilities |
| 37 | *E. coli* | 10 | *bla*_OXA-244_ | 2 | 1 isolate from a hospital and 1 sample taken by a GP |
| 38 | *E. coli* | 38 | *bla*_OXA-48_ | 7 | ≥2 healthcare facilities |
| 39 | *E. coli* | 38 | *bla*_OXA-48_ | 12 | ≥2 healthcare facilities |
| 40 | *E. coli* | 38 | *bla*_OXA-48_ | 4 | ≥2 healthcare facilities |
| 41 | *E. coli* | 38 | *bla*_OXA-48_ | 5 | ≥2 healthcare facilities |
| 42 | *E. coli* | 38 | *bla*_OXA-244_ | 3 | ≥2 healthcare facilities |
| 43 | *E. coli* | 38 | *bla*_OXA-48_ | 3 | 1 isolate from a hospital and 2 samples taken by a GP |
| 44 | *E. coli* | 38 | *bla*_OXA-48_ | 2 | 1 isolate from a hospital and 1 sample taken by a GP |
| 45 | *E. coli* | 69 | *bla*_OXA-244_ | 2 | 2 healthcare facilities |
| 46 | *E. coli* | 69 | *bla*_OXA-48_ | 2 | 2 samples taken by a GP |
| 47 | *E. coli* | 127 | *bla*_OXA-48_ | 10 | ≥2 healthcare facilities |
| 48 | *E. coli* | 131 | *bla*_VIM-1_ | 8 | 1 isolate from a hospital and 7 samples taken by a GP |
| 49 | *E. coli* | 167 | *bla*_NDM-5_ | 3 | ≥2 healthcare facilities |
| 50 | *E. coli* | 167 | *bla*_NDM-5_ | 2 | 2 samples taken by a GP |
| 51 | *E. coli* | 167 | *bla*_NDM-19_ | 2 | 1 isolate from a hospital and 1 sample taken by a GP |
| 52 | *E. coli* | 167 | *bla*_NDM-7_ | 3 | ≥2 healthcare facilities |
| 53 | *E. coli* | 167 | *bla*_NDM-5_ / *bla*_NDM-5_+*bla*_OXA-244_ | 2 (1 *bla*_NDM-5_ /  1 *bla*_NDM-5_+*bla*_OXA-244_) | 2 healthcare facilities |
| 54 | *E. coli* | 167 | *bla*_NDM-5_ | 2 | 2 healthcare facilities |
| 55 | *E. coli* | 227 | *bla*_OXA-48_ | 2 | 2 healthcare facilities |
| 56 | *E. coli* | 354 | *bla*_OXA-48_ | 3 | ≥2 healthcare facilities |
| 57 | *E. coli* | 361 | *bla*_NDM-5_ | 2 | 1 isolate from a hospital and 1 sample taken by a GP |
| 58 | *E. coli* | 405 | *bla*_NDM-5_ | 2 | 1 isolate from a hospital and 1 sample taken by a GP |
| 59 | *E. coli* | 405 | *bla*_NDM-5_ | 2 | 2 healthcare facilities |
| 60 | *E. coli* | 405 | *bla*_NDM-5_ | 2 | 1 isolate from a hospital and 1 sample taken by a GP |
| 61 | *E. coli* | 410 | *bla*_OXA-181_ / *bla*_NDM-5_ | 3 (2 *bla*_OXA-181_ / 1 *bla*_NDM-5_) | ≥2 healthcare facilities |
| 62 | *E. coli* | 410 | *bla*_NDM-5_ | 2 | 2 healthcare facilities |
| 63 | *E. coli* | 648 | *bla*_NDM-5_ | 2 | 1 isolate from a hospital and 1 sample taken by a GP |
| 64 | *E. coli* | 940 | *bla*_NDM-7_ | 2 | 2 healthcare facilities |
| 65 | *E. coli* | 1284 | *bla*_NDM-5_ | 2 | 1 isolate from a hospital and 1 sample taken by a GP |
| 66 | *E. coli* | 1598 | *bla*_OXA-48_ | 2 | 2 healthcare facilities |
| 67 | *E. coli* | 2851 | *bla*_OXA-181_ | 2 | 1 isolate from a hospital and 1 sample taken by a GP |
| 68 | *E. cloacae* complex | 66 | *bla*_OXA-48_ | 2 | 1 healthcare facility |
| 69 | *E. cloacae* complex | 78 | *bla*_OXA-48_ | 2 | 1 healthcare facility |
| 70 | *E. cloacae* complex | 78 | *bla*_VIM-1_ | 4 | ≥2 healthcare facilities |
| 71 | *E. cloacae* complex | 121 | *bla*_OXA-48_ | 8 | ≥2 healthcare facilities |
| 72 | *E. cloacae* complex | 591 | No carbapenemase-encoding gene found | 2 | 1 isolate from a hospital and 1 sample taken by a GP |
| 73 | *E. cloacae* complex | 928 | *bla*_VIM-1_ | 2 | 1 healthcare facility |
| 74 | *E. cloacae* complex | 78 / 1193 | *bla*_OXA-48_ | 11 (10 ST78 / 1 ST1193) | ≥2 healthcare facilities |
| 75 | *C. freundii* | 19 | *bla*_OXA-514_ | 3 | ≥2 healthcare facilities |
| 76 | *C. freundii* | 22 | *bla*_NDM-5_ / *bla*_NDM-5_+*bla*_OXA-48_ | 38 (37 *bla*_NDM-5_ /  1 *bla*_NDM-5_+*bla*_OXA-48_) | ≥2 healthcare facilities |
| 77 | *C. freundii* | 259 | *bla*_OXA-48_ | 2 | 2 healthcare facilities |

CPE: carbapenemase-producing Enterobacterales; GP: general practitioner; ST: sequence type; WGS: Whole genome sequencing.
